# Information

**Published:** 1898-11

**Authors:** 


### Information.

A Magazine for the Dental Office Reception-room Table.

This is a monthly journal devoted to hygiene and general information of interest to dentists, and people generally. Edited and published by Dr. L. P. Bethel, Kent, Ohio.

The object of this new magazine is to instruct the public regarding Oral Hygiene, and gives useful information to the dentist.

This enterprise is one that will be of decided advantage to both the dentist and the public, and especially the latter, if properly conducted, as we have no doubt lt will be under the management of Dr. Bethel, who is certainly well equipped for an enterprise of this kind, having been engaged for years in editorial work and in teaching, writing, and general practice as well.

The question of information in regard to diseases of the teeth and mouth and other influences, and the possibilities in dental practice for remedying these conditions has been a question of much consideration in bygone years. Questions involved in it have not been very satisfactorily decided. This enterprise certainly looks more definitely to a solution of the matter than anything that has been attempted hitherto. The intent is to make the "Journal" a vehicle of general information to everyone, and especially to parents who have charge of children, to teachers, and the young as well.

In order to meet all these requirements will require peculiar ability, and not of one man alone. The co-operation of quite a number of writers in the profession has been secured for this work, and we would suggest and urge that the co-operation of every dentist who desires the elevation of his profession should be given to this work.

It is issued monthly at $1.00 per year.

